# Sustained effect of habitual feeding time on daily rhythm of core body temperature in mice

**DOI:** 10.3389/fnut.2022.966788

**Published:** 2022-08-22

**Authors:** Hitoshi Ando, Naoto Nagata, Takashi Hosono, Nazmul Hasan, Jun-ichi Morishige, Takiko Daikoku, Yoshiko Maida, Masanori Ono, Tomoko Fujiwara, Hiroshi Fujiwara

**Affiliations:** ^1^Department of Cellular and Molecular Function Analysis, Graduate School of Medical Sciences, Kanazawa University, Kanazawa, Japan; ^2^Department of Obstetrics and Gynecology, Graduate School of Medical Sciences, Kanazawa University, Kanazawa, Japan; ^3^Division of Animal Disease Model, Research Center for Experimental Modeling of Human Disease, Kanazawa University, Kanazawa, Japan; ^4^Faculty of Health Sciences, Institute of Medical, Pharmaceutical and Health Sciences, Kanazawa University, Kanazawa, Japan; ^5^Department of Obstetrics and Gynecology, Tokyo Medical University, Tokyo, Japan; ^6^Department of Human Life Environments, Kyoto Notre Dame University, Kyoto, Japan

**Keywords:** core body temperature (CBT), diet-induced thermogenesis (DIT), circadian rhythm, meal timing, skipping breakfast

## Abstract

**Background and aim:**

Circadian clocks in most peripheral tissues are entrained mainly by feeding. Therefore, this study aimed to investigate whether the daily rhythm of core body temperature (CBT), including the effect of diet-induced thermogenesis, varies according to habitual feeding time.

**Methods:**

Wild-type and uncoupling protein 1 (UCP1) knockout mice were fed only during the first 4 h (Breakfast group) or the last 4 h of the dark period (Dinner group) for 17 days. On day 18, both groups were fed twice for 2 h, at the same starting times. Locomotor activity and CBT were measured continuously during the experiment.

**Results:**

On day 18, CBT increased at the beginning of each feeding period, regardless of the group and strain. However, the CBT increase induced by the first meal decreased sharply in the Breakfast group and mildly in the Dinner group; the opposite was observed after the second meal. In UCP1 knockout, but not wild-type, mice, the total amount of CBT was significantly lower in the Dinner group than in the Breakfast group. These effects were mostly independent of the locomotor activity and food intake.

**Conclusion:**

These results reveal that the effect of habitual feeding time on the daily rhythm of CBT is sustained at least until the following day. These effects may be mediated by both UCP1-dependent and -independent mechanisms.

## Introduction

Body temperature is well-known to exhibit daily variations. Because both physical activity and feeding induce thermogenesis, body temperature usually rises during the active phase (day for humans and night for most rodents) (1). In addition, diet-induced thermogenesis (DIT), facultative thermogenesis in response to single meals (2, 3), is reported to be higher in the morning than in the evening in humans (4–6). This is a putative mechanism underlying the association between meal timing and weight status. Specifically, skipping breakfast and/or eating at night may lead to obesity due to decreased DIT (7, 8). Daily rhythms in many behavioral and physiological processes are generated by circadian clocks, which are composed of transcriptional/translational feedback loops involving a set of clock genes (9). In mammals, the suprachiasmatic nuclei of the hypothalamus possess the central clock, whereas almost all other tissues have so-called peripheral clocks, which share the same machinery as the central clock. Recently, accumulating evidence has indicated an important role of peripheral clocks (10). For example, studies using mice with tissue-specific deletion of *Bmal1*, a core clock gene, have revealed that hepatic glucose production and muscular glucose metabolism are regulated by peripheral clocks in their respective tissues (11, 12). Moreover, we recently found that the circadian clock in brown adipose tissue (BAT) regulates fatty acid utilization and thermogenesis in BAT (13). Collectively, these findings suggest that the circadian rhythmicity of body temperature is directly regulated by peripheral clocks.

The central clock is regulated by light stimuli and communicates this information to the peripheral clock through neural, endocrine, temperature, and behavioral signals (10). Consequently, the phases of the circadian clock in most peripheral tissues are mainly set by feeding instead of light (14, 15). This raises the possibility that habitual meal timing affects the daily rhythm of body temperature, including the effect of DIT. For instance, DIT might be lower in the morning than in the afternoon among habitual breakfast skippers. However, this hypothesis has not yet been thoroughly tested. To address this issue, we investigated whether the daily profile of core body temperature (CBT) differs depending on the habitual feeding time, even during the same feeding schedule.

## Materials and methods

### Animals

Male C57BL/6J mice (*n* = 15) at 6 weeks of age were obtained from Japan SLC (Hamamatsu, Japan). Mitochondrial uncoupling protein 1 (UCP1) knockout (KO) mice with a C57BL/6J background (16) were originally purchased from Jackson Laboratory (Bar Harbor, ME, United States; stock No. 003124) and bred at Kanazawa University (Kanazawa, Japan). Male mice (*n* = 8) were used in this study. All mice were fed a regular diet (CRF-1, Oriental Yeast, Tokyo, Japan) and maintained under controlled temperature (∼23°C) and light (12-h/12-h light/dark cycle) conditions.

### Experimental design

Zeitgeber time (ZT) is used to describe the experimental time, with ZT 0 defined as lights on and ZT 12 as lights off.

**FIGURE 1:**
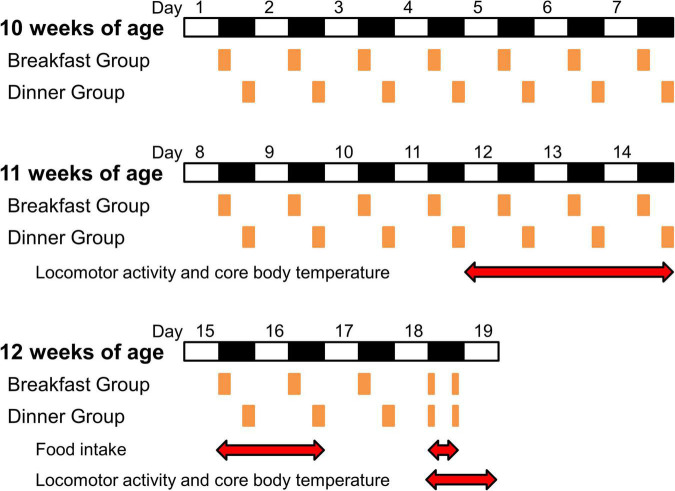
Protocol for the experiments. White and black bars indicate light and dark periods, respectively. Orange boxes indicate feeding times. Red double-headed arrows show measurement periods for analysis.

At 10 weeks of age (day 1), wild-type C57BL/6J mice were divided into three groups (*n* = 5/each group): Ad lib group (fed chow *ad libitum*), Breakfast group (in which feeding time was restricted to the first 4 h of the dark period [ZT 12–16]), and Dinner group (in which feeding time was restricted to the last 4 h of the dark period [ZT 20–24]). UCP1 KO mice were divided into the Breakfast and Dinner groups (*n* = 4/each group). On day 18, both the Breakfast and Dinner groups were fed chow twice for 2 h during ZT 12–14 and ZT 20–22 (Figure 1).

To reduce the effects of external stimuli other than light and feeding, we did not open the sound-attenuating chamber during days 9–14, and the data of locomotor activity and CBT during the last 3 days (days 12–14) were used for analysis (Figure 1). Thereafter, food intake was manually measured on days 15, 16, and 18.

### Measurements of core body temperature and locomotor activity

At 8 weeks of age, all mice were implanted intraperitoneally with an ultra-small temperature logger (DST Nano-T, Star-Oddi, Gardabaer, Iceland) under anesthesia. The temperature resolution and accuracy of this logger were 0.032°C and ±0.2°C, respectively. CBT was continuously measured at 5 minute intervals until the end of the experiment. Mercury software (version 5.99, Star-Oddi) was used to program the loggers and to retrieve the stored data.

At 2 weeks after logger implantation, the mice were transferred to specialized cages (*n* = 1/cage) for infrared sensor detection and feeding time control (Supermex, Muromachi Kikai, Tokyo, Japan), each of which was placed in a sound-attenuating chamber (Muromachi Kikai). Locomotor activity was continuously measured at 5 minute intervals until the end of the experiment. CompACT AMS software (version 3.86, Muromachi Kikai) was used to store and analyze the data.

### Statistical analysis

Data are presented as the mean and standard deviation (SD) or median. Differences between groups were analyzed using Student’s *t*-test, Mann-Whitney test, or one-way analysis of variance (ANOVA) followed by the Bonferroni *post-hoc* test. Calculations were performed using SPSS 24.0 (IBM SPSS Statistics, Chicago, IL, United States). *P*-value < 0.05 was considered significant.

## Results

### Effects of habitual feeding time on a routine day

**FIGURE 2:**
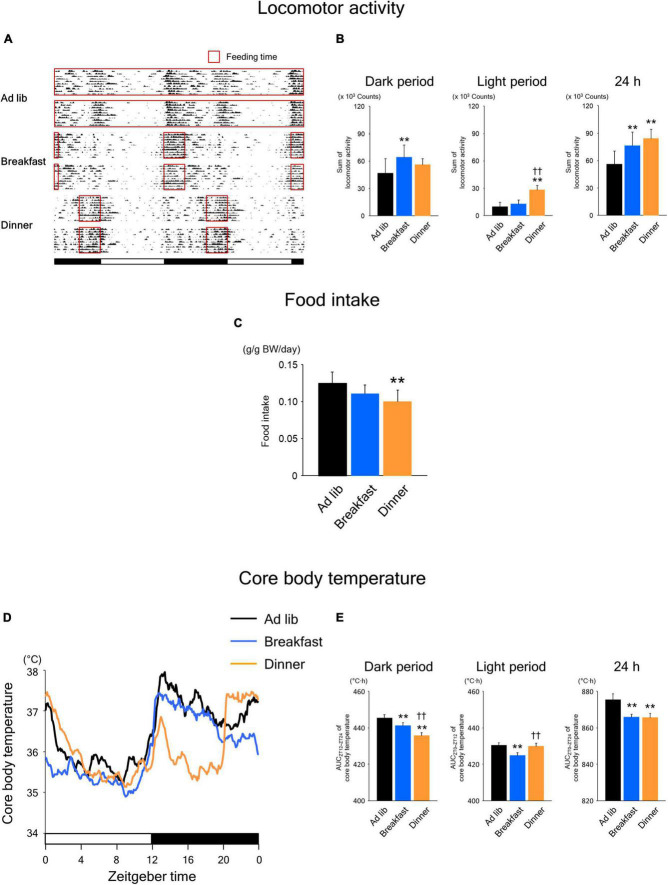
Effects of time-restricted feeding on locomotor activity **(A,B)**, food intake **(C)**, and core body temperature (CBT) **(D,E)** in wild-type mice. **(A)** Representative double-plotted actograms of locomotor activity during days 8–18 (*n* = 2 for each group). Red open boxed indicate feeding periods. Bottom white and black bars indicate light and dark periods, respectively. **(B)** Amounts of locomotor activity during the dark period, light period, and the total 24-h period. Data are means + SD of 15 values obtained from five mice. **P < 0.01 vs. Ad lib; ^††^*P* < 0.01 vs. Breakfast. **(C)** Daily food intake. Data are means + SD of 10 values obtained from five mice. **P < 0.01 vs. Ad lib. **(D)** Daily profiles of CBT. Data are means of 15 values obtained from five mice. **(E)** Area under the curve (AUC) of CBT during the dark period, light period, and the total 24-h period. Data are means + SD of 15 values obtained from five mice. **P < 0.01 vs. Ad lib; ^††^*P* < 0.01 vs. Breakfast.

As shown in Figure 2A, the wild-type mice were active during the dark period. In the Ad lib group, locomotor activity was biphasic, peaking at the beginning and end of the dark period. On the other hand, activity increased monophasically during the respective feeding period in both the Breakfast and Dinner groups. Interestingly, the total 24-h locomotor activity was significantly higher in both the Breakfast and Dinner groups than in the Ad lib group (Figure 2B). In particular, the dark-period amount increased in the Breakfast group, whereas the light-period amount increased in the Dinner group. As for food intake, the Dinner group, but not the Breakfast group, consumed less chow than the Ad lib group (Figure 2C).

Habitual feeding time also affected the daily rhythm of CBT (Figure 2D). In the Breakfast group, similar to the Ad lib group, the CBT increased at the beginning of the dark period and remained high during the middle of the dark period. Meanwhile, the increase around the end of the active phase, which was observed in the Ad lib group, disappeared in the Breakfast group. In contrast, in the Dinner group, the increase at the beginning of the active phase was small and transient, and CBT was markedly lower during ZT 16–20 and similarly high around ZT 0, compared to the Ad lib group. Consequently, the area under the curve (AUC) of CBT during the dark period was significantly lower in both the Breakfast and Dinner groups than in the Ad lib group, while the AUC during the light period was lower in Breakfast group than in the other groups (Figure 2E). However, the AUC of CBT during the 24 h period was equally decreased in both the Breakfast and Dinner groups. These results are not surprising because the differences in daily CBT profiles among the groups may be explained largely by DIT and the thermic effect of physical activity.

### Sustained effects of habitual feeding time on the following day

**FIGURE 3:**
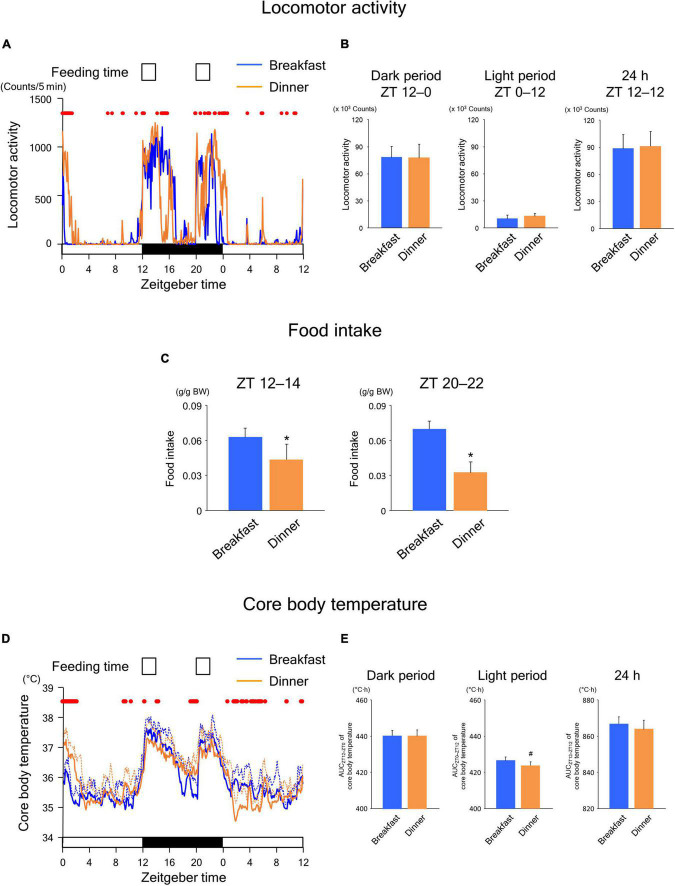
Sustained effects of time-restricted feeding on locomotor activity **(A,B)**, food intake **(C)**, and CBT **(D,E)** on days 18–19 in wild-type mice. **(A)** Daily profiles of locomotor activity. Data are medians of five mice. Red dots indicate *P* < 0.05 between the groups at each time point. **(B)** Amounts of locomotor activity during the dark period on day 18, light period on day 19, and the total 24-h period. Data are means + SD of five mice. **(C)** Food intake. Data are means + SD of five mice. *P < 0.05. **(D)** Daily profiles of CBT. Solid and dotted lines indicate means and means + SD, respectively, of five mice. Red dots indicate *P* < 0.05 between the groups at each time point. **(E)** AUC of CBT during the dark period on day 18, light period on day 19, and the total 24-h period. Data are means + SD of five mice. ^#^*P* < 0.1.

To explore whether the effects of time-restricted feeding continued until the following day, we applied the same feeding schedule (twice for 2 h each, during ZT 12–14 and ZT 20–22) for both the Breakfast and Dinner groups on day 18. As shown in Figure 3A, locomotor activity increased biphasically around the same time as feeding in both groups. In addition, the amounts during both dark and light periods were comparable between the groups (Figure 3B). However, the increased activity during feeding seemed to continue for a longer period in the group that had been active during the same time frame on the previous days (Figure 2A); the Breakfast group was more active just after the first meal, whereas the Dinner group was more active just after the second meal (Figure 3A). The difference in food intake between groups was more evident; the Dinner group consumed less chow than the Breakfast group during both ZT 12–14 and ZT 20–22 (Figure 3C). These results indicated that the effects of habitual feeding time on locomotor activity and appetite were sustained until the following day.

In the Breakfast group, CBT was elevated at the beginning of the dark period and thereafter decreased substantially during the middle of the dark period (Figure 3D). However, the decrease following the first meal was mild in the Dinner group. In contrast, the increase in CBT during the second meal was reduced during ZT 0–4 to a greater extent in the Dinner group than in the Breakfast group. This difference in CBT profiles between groups cannot be accounted for by differences in the amounts of physical activity and/or food intake. Taken together, these results strongly suggest that daily rhythmicity in the thermic response to a meal, including the DIT, is also affected by habitual feeding time. Interestingly, the AUC of CBT was not significantly different between groups, although the AUC during the light period tended to be lower in the Dinner group than in the Breakfast group (Figure 3E). This indicates that the Dinner group required less chow to produce a comparable DIT to that of the Breakfast group.

### Sustained effects of habitual feeding time in UCP1 KO mice

It has been shown that BAT is involved in DIT (17, 18), and UCP1, the protein responsible for the thermogenic process in BAT, contributes to DIT (19). If the above findings observed in wild-type mice resulted from a change in the daily rhythm of UCP1-dependent thermogenesis, the difference in CBT profiles between groups ought to disappear in UCP1 KO mice. Therefore, we investigated the effects of time-restricted feeding in UCP1 KO mice.

**FIGURE 4:**
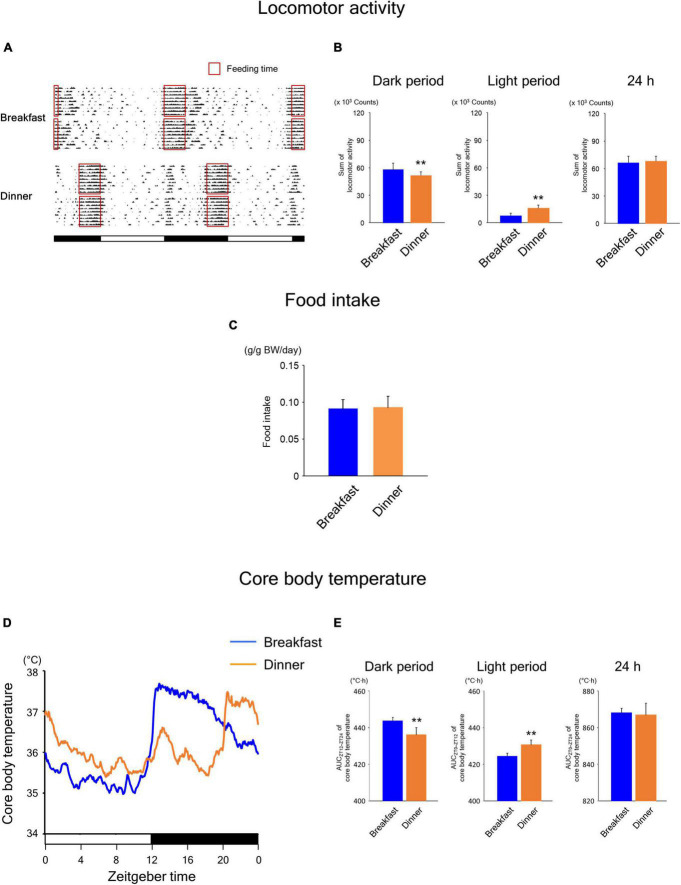
Effects of time-restricted feeding on locomotor activity **(A,B)**, food intake **(C)**, and CBT **(D,E)** in uncoupling protein 1 (UCP1) knockout (KO) mice. **(A)** Representative double-plotted actograms of locomotor activity during days 9–18 (*n* = 2 for each group). Red open boxed indicate feeding periods. Bottom white and black bars indicate light and dark periods, respectively. **(B)** Amounts of locomotor activity during the dark period, light period, and the total 24-h period. Data are means + SD of 12 values obtained from four mice. **P < 0.01. **(C)** Daily food intake. Data are means + SD of 8 values obtained from four mice. **(D)** Daily profiles of CBT. Data are means of 12 values obtained from four mice. **(E)** AUC of CBT during the dark period, light period, and the total 24-h period. Data are means + SD of 12 values obtained from four mice. **P < 0.01.

During the time-restricted feeding period, the daily rhythms of locomotor activity (Figures 4A,B) were similar to those in wild-type mice (Figures 2A,B). However, unlike in wild-type mice (Figure 2C), food intake was comparable between the Breakfast and Dinner groups in UCP1 KO mice (Figure 4C). The daily rhythms of CBT (Figures 4D,E) were similar to those in wild-type mice (Figures 2D,E). Interestingly, the levels of CBT after feeding were comparable to those in wild-type mice, suggesting that CBT can be sufficiently increased after feeding even without UCP1.

**FIGURE 5:**
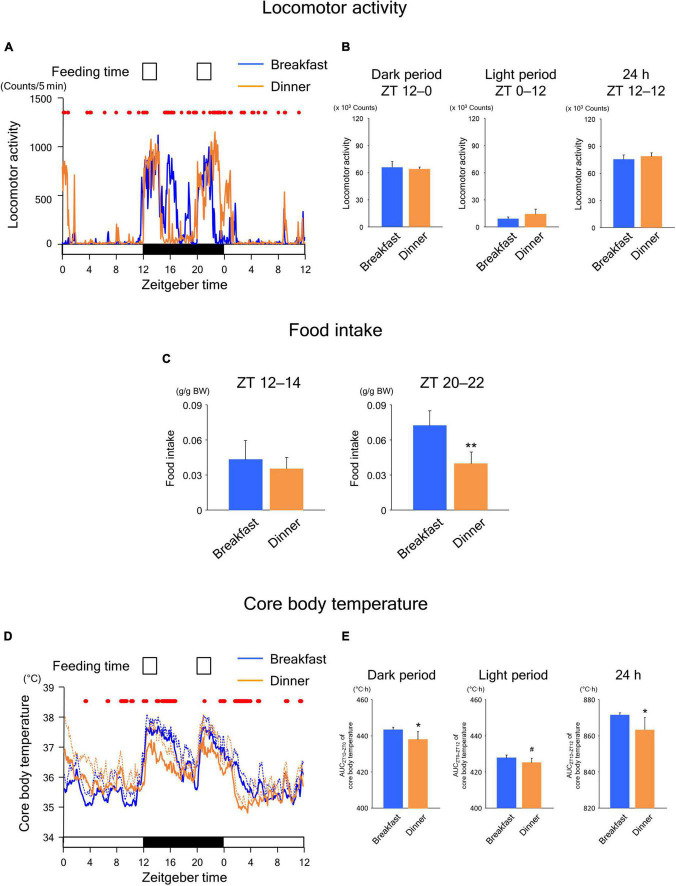
Sustained effects of time-restricted feeding on locomotor activity **(A,B)**, food intake **(C)**, and CBT **(D,E)** on days 18–19 in UCP1 KO mice. **(A)** Daily profiles of locomotor activity. Data are medians of four mice. Red dots indicate *P* < 0.05 between the groups at each time point. **(B)** Amounts of locomotor activity during the dark period on day 18, light period on day 19, and the total 24-h period. Data are means + SD of four mice. **(C)** Food intake. Data are means + SD of four mice. **P < 0.01. **(D)** Daily profiles of CBT. Solid and dotted lines indicate means and means + SD, respectively, of four mice. Red dots indicate *P* < 0.05 between the groups at each time point. **(E)** AUC of CBT during the dark period on day 18, light period on day 19, and the total 24-h period. Data are means + SD of four mice. ^#^*P* < 0.1, *P < 0.05.

Similar to the findings in wild-type mice, the Breakfast group was more active around ZT 16 on day 18, whereas the Dinner group was more active around ZT 0 on day 19 (Figure 5A). Locomotor activity during both dark and light periods was comparable between the groups (Figure 5B). In addition, although the food intake of the first meal did not differ between the groups, that of the second meal was significantly lower in the Dinner group than in the Breakfast group (Figure 5C). Thus, the effects of habitual feeding on locomotor activity and appetite were sustained also in UCP1 KO mice.

As shown in Figure 5D, the daily CBT profiles were similar to those observed in wild-type mice. However, the AUC values of CBT during both the dark and 24-h periods were significantly lower in the Dinner group than in the Breakfast group (Figure 5E), unlike the findings in wild-type mice (Figure 3E). Because these effects were mostly independent of locomotor activity and food intake, our data revealed that habitual feeding time affects the daily rhythms of both UCP1-dependent and -independent thermogenesis.

## Discussion

The present study revealed for the first time, to our knowledge, that habitual feeding time influences the daily rhythm on CBT even during the same feeding schedule. Specifically, habitual feeding time significantly affected the CBT response to food intake. In addition, this effect could not be explained by the differences in the amounts of physical activity and/or food intake. Taken together, these results suggest that habitual feeding time affects the daily rhythm of DIT.

In mice, DIT was reported to fully emanate from UCP1 activity in BAT (19, 20). However, later studies revealed that DIT is also mediated by sarcolipin, a regulator of sarcoplasmic/endoplasmic reticulum Ca^2+^ ATPase (SERCA) activity in skeletal muscles (21, 22). Interestingly, UCP1 gene expression is regulated by the clock gene Reverbα and, therefore, exhibits circadian rhythmicity in BAT (23). In addition, many genes encoding proteins involved in Ca^2+^ signaling are circadianly expressed in the skeletal muscle, and at least a subset of them, including the SERCA gene, may be regulated by the core clock component CLOCK-BMAL1 (24, 25). Furthermore, we have shown that mice with brown adipocyte-specific *Bmal1* KO show reduced thermogenesis despite the elevated expression of UCP1, probably due to impaired fatty acid utilization in BAT (13). Thus, thermogenesis in BAT and skeletal muscle may be directly regulated by the respective intracellular clock. Therefore, it seemed reasonable that habitual feeding time affects the “circadian” (i.e., endogenous 24-h) rhythm of both UCP1-dependent and –independent DIT.

Unexpectedly, however, the profiles of CBT after meals on day 18 appeared to be opposite to those during the previous days in both the Breakfast and Dinner groups. Specifically, in the Breakfast group, the CBT increase induced by feeding lasted until the end of the dark period during once-daily time-restricted feeding (Figure 2D) but disappeared in the middle of the dark period during the first meal on day 18 (Figure 3D). Similar findings were observed for CBT during ZT 0–4 on day 19 in the Dinner group. Additionally, UCP1 KO mice also showed a sharp decrease in CBT during both ZT 16–20 on day 18 in the Breakfast group and ZT 0–4 on day 19 in the Dinner group (Figure 5D). Given that DIT is the heat produced in response to excess energy intake (2), these results could be explained by the circadian rhythm of resting metabolic rate (RMR). The RMR consists of two components: the standard metabolic rate and the thermic effect of food. The former is defined as the amount of energy at rest in a thermoneutral environment, and the latter as the heat generated during the digestion, absorption, and processing of food (2). Circadian clocks regulate a broad spectrum of physiological functions including metabolism, digestion, and absorption of food (26). Consequently, RMR exhibits a circadian rhythm with a peak in the early evening in humans (27). Interestingly, a recent study reported that the apparent difference in DIT between the morning and evening could be explained by the circadian variation of RMR in healthy subjects (28). Because energy expenditure was not measured in this study, future studies are needed to determine whether the circadian rhythm of RMR is involved in the sustained effects of habitual feeding time on the daily CBT profile.

In wild-type mice, the Breakfast and Dinner groups exhibited comparable AUC values of CBT although food intake was significantly lower in the Dinner group than in the Breakfast group (Figure 3). In UCP1 KO mice, however, CBT increase after the first meal was significantly lower in the Dinner group than in the Breakfast group although food intake was comparable (Figure 5). These results indicate that DIT induced by the first meal is highly UCP1-dependent in the Dinner group. When extrapolated to humans, the results of this study raise a new question: is it better for habitual breakfast skippers to have breakfast occasionally? If UCP1 activity is low, occasional breakfast consumption might induce inadequate thermogenesis and lead to energy excess and body weight gain. Recently, Guinter et al. reported that women who reported eating breakfast occasionally (3–4 days/week) were significantly more likely to be obese than women who always ate breakfast (7 days/week) and those who never ate breakfast (<1 day/week) (29). Moreover, women who rarely consumed breakfast (1–2 days/week) were also more likely to be obese than those who never consumed breakfast, although the study did not compare them directly. These results can be explained, at least in part, by the disruption of the peripheral clocks. However, the phase shift of the RMR rhythm induced by habitual breakfast skipping may also be involved in the relationship between regularity in breakfast consumption and weight status. Further studies are required to verify this hypothesis.

## Conclusion

This study clearly indicates that the effect of habitual feeding time on the daily rhythm of CBT is sustained, at least until the following day, independent of the effects of physical activity and feeding. These effects may be mediated by both UCP1-dependent and -independent mechanisms. Future studies should evaluate whether and how much this effect contributes to health outcomes.

## Data availability statement

The raw data supporting the conclusions of this article will be made available by the authors, without undue reservation.

## Ethics statement

This animal study was reviewed and approved by the Institutional Committee for the Ethical Use of Experimental Animals (Approval No. AP-163792) and performed in accordance with the Guidelines for the Care and Use of Laboratory Animals at Kanazawa University (Kanazawa, Japan).

## Author contributions

HA conceptualized the study, designed the research, performed the experiments, analyzed the data, and co-wrote the manuscript with input from all co-authors. NN, TH, NH, and J-iM performed the experiments. TD, YM, MO, TF, and HF provided expertise and resources. All authors contributed to the article and approved the submitted version.

## References

[1] Refinetti R. The circadian rhythm of body temperature. *Front Biosci.* (2010) 15:564–94. 10.2741/3634 20036834

[2] Tran LT Park S Kim SK Lee JS Kim KW Kwon O. Hypothalamic control of energy expenditure and thermogenesis. *Exp Mol Med.* (2022) 54:358–69. 10.1038/s12276-022-00741-z 35301430 PMC9076616

[3] Saito M Matsushita M Yoneshiro T Okamatsu-Ogura Y. Brown adipose tissue, diet-induced thermogenesis, and thermogenic food ingredients: from mice to men. *Front Endocrinol.* (2020) 11:222. 10.3389/fendo.2020.00222 32373072 PMC7186310

[4] Romon M Edme JL Boulenguez C Lescroart JL Frimat P. Circadian variation of diet-induced thermogenesis. *Am J Clin Nutr.* (1993) 57:476–80. 10.1093/AJCN/57.4.476 8460600

[5] Bo S Fadda M Castiglione A Ciccone G De Francesco A Fedele D Is the timing of caloric intake associated with variation in diet-induced thermogenesis and in the metabolic pattern? A randomized cross-over study. *Int J Obes.* (2015) 39:1689–95. 10.1038/IJO.2015.138 26219416

[6] Richter J Herzog N Janka S Baumann T Kistenmacher A Oltmanns KM. Twice as high diet-induced thermogenesis after breakfast vs dinner on high-calorie as well as low-calorie meals. *J Clin Endocrinol Metab.* (2020) 105:211–21. 10.1210/clinem/dgz311 32073608

[7] Davis R Rogers M Coates AM Leung GKW Bonham MP. The impact of meal timing on risk of weight gain and development of obesity: a review of the current evidence and opportunities for dietary intervention. *Curr Diab Rep.* (2022) 22:147–55. 10.1007/s11892-022-01457-0 35403984 PMC9010393

[8] Shaw E Leung GKW Jong J Coates AM Davis R Blair M The impact of time of day on energy expenditure: implications for long-term energy balance. *Nutrients.* (2019) 11:2383. 10.3390/nu11102383 31590425 PMC6835928

[9] Takahashi JS. Transcriptional architecture of the mammalian circadian clock. *Nat Rev Genet.* (2017) 18:164–79. 10.1038/nrg.2016.150 27990019 PMC5501165

[10] Stenvers DJ Scheer FAJLJL Schrauwen P la Fleur SE Kalsbeek A Jan Stenvers D. Circadian clocks and insulin resistance. *Nat Rev Endocrinol.* (2018) 15:75–89. 10.1038/s41574-018-0122-1 30531917

[11] Ando H Ushijima K Shimba S Fujimura A. Daily fasting blood glucose rhythm in male mice: a role of the circadian clock in the liver. *Endocrinology.* (2016) 157:463–9. 10.1210/en.2015-1376 26653333

[12] Dyar KA Ciciliot S Wright LE Biensø RS Tagliazucchi GM Patel VR Muscle insulin sensitivity and glucose metabolism are controlled by the intrinsic muscle clock. *Mol Metab.* (2014) 3:29–41. 10.1016/j.molmet.2013.10.005 24567902 PMC3929910

[13] Hasan N Nagata N Morishige JI Islam MT Jing Z Harada KI Brown adipocyte-specific knockout of Bmal1 causes mild but significant thermogenesis impairment in mice. *Mol Metab.* (2021) 49:101202. 10.1016/j.molmet.2021.101202 33676029 PMC8042177

[14] Damiola F Le Minli N Preitner N Kornmann B Fleury-Olela F Schibler U. Restricted feeding uncouples circadian oscillators in peripheral tissues from the central pacemaker in the suprachiasmatic nucleus. *Genes Dev.* (2000) 14:2950–61. 10.1101/gad.183500 11114885 PMC317100

[15] Hosono T Ono M Daikoku T Mieda M Nomura S Kagami K Time-restricted feeding regulates circadian rhythm of murine uterine clock. *Curr Dev Nutr.* (2021) 5:nzab064. 10.1093/cdn/nzab064 33981944 PMC8099714

[16] Enerbäck S Jacobsson A Simpson EM Guerra C Yamashita H Harper M-E Mice lacking mitochondrial uncoupling protein are cold-sensitive but not obese. *Nature.* (1997) 387:90–4. 10.1038/387090a0 9139827

[17] Hibi M Oishi S Matsushita M Yoneshiro T Yamaguchi T Usui C Brown adipose tissue is involved in diet-induced thermogenesis and whole-body fat utilization in healthy humans. *Int J Obes.* (2016) 40:1655–61. 10.1038/ijo.2016.124 27430878 PMC5116053

[18] Din MU Saari T Raiko J Kudomi N Maurer SF Lahesmaa M Postprandial oxidative metabolism of human brown fat indicates thermogenesis. *Cell Metab.* (2018) 28:207–16.e3. 10.1016/j.cmet.2018.05.020 29909972

[19] Feldmann HM Golozoubova V Cannon B Nedergaard J. UCP1 ablation induces obesity and abolishes diet-induced thermogenesis in mice exempt from thermal stress by living at thermoneutrality. *Cell Metab.* (2009) 9:203–9. 10.1016/j.cmet.2008.12.014 19187776

[20] von Essen G Lindsund E Cannon B Nedergaard J. Adaptive facultative diet-induced thermogenesis in wild-type but not in UCP1-ablated mice. *Am J Physiol Endocrinol Metab.* (2017) 313:E515–27. 10.1152/ajpendo.00097.2017 28679625

[21] Bal NC Maurya SK Sopariwala DH Sahoo SK Gupta SC Shaikh SA Sarcolipin is a newly identified regulator of muscle-based thermogenesis in mammals. *Nat Med.* (2012) 18:1575–9. 10.1038/nm.2897 22961106 PMC3676351

[22] Rowland LA Maurya SK Bal NC Kozak L Periasamy M. Sarcolipin and uncoupling protein 1 play distinct roles in diet-induced thermogenesis and do not compensate for one another. *Obesity.* (2016) 24:1430–3. 10.1002/oby.21542 27238087 PMC4925282

[23] Gerhart-Hines Z Feng D Emmett MJ Everett LJ Loro E Briggs ER The nuclear receptor Rev-erbα controls circadian thermogenic plasticity. *Nature.* (2013) 503:410–3. 10.1038/nature12642 24162845 PMC3839416

[24] McCarthy JJ Andrews JL McDearmon EL Campbell KS Barber BK Miller BH Identification of the circadian transcriptome in adult mouse skeletal muscle. *Physiol Genomics.* (2007) 31:86–95. 10.1152/physiolgenomics.00066.2007 17550994 PMC6080860

[25] Cavieres-Lepe J Ewer J. Reciprocal relationship between calcium signaling and circadian clocks: implications for calcium homeostasis, clock function, and therapeutics. *Front Mol Neurosci.* (2021) 14:666673. 10.3389/fnmol.2021.666673 34045944 PMC8144308

[26] Tahara Y Shibata S. Chronobiology and nutrition. *Neuroscience.* (2013) 253:78–88. 10.1016/j.neuroscience.2013.08.049 24007937

[27] Zitting KM Vujovic N Yuan RK Isherwood CM Medina JE Wang W Human resting energy expenditure varies with circadian phase. *Curr Biol.* (2018) 28:3685–90.e3. 10.1016/j.cub.2018.10.005 30416064 PMC6300153

[28] Ruddick-Collins LC Flanagan A Johnston JD Morgan PJ Johnstone AM. Circadian rhythms in resting metabolic rate account for apparent daily rhythms in the thermic effect of food. *J Clin Endocrinol Metab.* (2022) 107:E708–15. 10.1210/clinem/dgab654 34473293 PMC8764350

[29] Guinter MA Park YM Steck SE Sandler DP. Day-to-day regularity in breakfast consumption is associated with weight status in a prospective cohort of women. *Int J Obes.* (2020) 44:186–94. 10.1038/S41366-019-0356-6 30926951 PMC6766424

